# A Treatise on Corns, Bunions, the Diseases of the Nails, and the General Management of the Feet

**Published:** 1845-04

**Authors:** 


ART. VII.—*A Treatise on Corns, Bunions, the Diseases of the Nails, and the general management of the Feet.* By LEWIS DURLACHER, Surgeon Chiropodist (by special appointment) to the Queen.—*London*, 1845. 8vo, pp. 196.

WE suspect that medical men, in general, are disposed greatly to underrate the importance of the diseases treated of in this book, in their bearings both on hygiene and therapeutics. When, however, it is considered how essential *exercise* is to the promotion of health, and how necessary it is in the treatment of many chronic diseases, any obstacle to the best form of exercise, *walking*, must be allowed to be a matter of the very first consequence. How sadly *corns* interfere with walking everybody knows. "These little things are great to little men." Mr. Durlacher's treatise ought, therefore, to be received by the profession, not merely as suplying the best information to enable them or their friends to relieve immediate suffering, but as ministering to general therapeutics, in one of its highest aspects. It is a modest and sensible performance, the result obviously of much experience, and written in a fair and honest spirit. It is, in fact, the only work on the subject which has any pretensions to scientific accuracy, or can claim the attention of the legitimate medical practitioner.

The following statement shows the spirit in which Mr. Durlacher's book is composed, and shows that it may be safely recommended to our patients:

"Although I have devoted nearly thirty years' practical experience to the investigation, and have tried various chemical and other remedial agents, yet I have never been able to disover any certain cure for corns. Nevertheless, men are occasionally to be found bold enough in their ignorance and presumption, to assert by public advertisement, that they possess an infallible nostrum, capable of thoroughly eradicating corns; and others, who pretend to extract them, seek to aid their trickey and charlatanerie by exhibiting small spicula as the roots of the corns they have extracted, although it is a positive fact, from the structure of the skin, that such an assertion must be false, and the whole proceeding the veriest imposition imaginable." (Preface, p. x.)

The external application in which the author most confides, and which he principally employs, is nitrate of silver. Our own experience had long convinced us of its pre-eminent efficacy. The volume contains many things deserving notice, but our waning space forbids our embracing them. At p. 49, the author describes "a kind of neuralgia seated between the toes," accompanied with severe pain, but for which, on examination, no obvious cause, (that is, no corn or callosity,) can be discovered. It is not a common complaint, but it is useful to know that it occasionally occurs. Like affections of the nerves in general, it sometimes defies all treatment, which, to have any chance of success, must be general as well as local.

Another case is worthy of notice: it is one in which Mr. Durlacher's diagnosis gave a happy solution in circumstances of some delicacy. A young gentleman was confined by illness, and thinking this a convenient opportunity for having a troublesome corn extracted, sent for the author. Mr. Durlacher found on the second joint of the third toe, "a festered corn, attended with inflammation," the foot being swollen and red streaks passing upwards towards the leg. The author inquired of the patient if he had any pain in the groin? "The question," says Mr. Durlacher, "seemed to astonish him, as he was confined solely on that account, and the swelling was attributed by his medical attendent, to a cause which he (the patient) considered could not possibly be the case." (p. 60.) Mr. Durlacher speedily relieved the patient's anxieties as to the source of the original pain; the corn was extracted; fetid serum was discharged; lunar caustic, hot fomentations, and a linseed-meal poultice were successively and successfully applied, the pain and swelling in the groin now subsiding.

The author states that the volume is intended for general readers as well as for the profession; and its freedom from technical language, and the perspicuity of the directions which it gives, adapt it for this purpose.

